# On the Means of Ascertaining Infanticide

**Published:** 1826-07

**Authors:** 


28. *Infanticide.* Every one is aware of the amount of prejudice which obtains on the Bench, and timidity or doubt in the witness-box, on the subject of tests respecting the viviparous or still-born condition of an infant, whose mother lies under the suspicion of infanticide. We are disposed to agree with some late medico-legal writers, especially Dr. Smith, that the proper tests, when carefully applied, will very generally point out the true state of things. But as, unfortunately, there are exceptions to the general rule, the awful responsibility on the part of the medical witness, and the prejudice which is acknowledged to exist at the Bar, will always lead the physician or surgeon to hesitate in giving a very decided opinion, even when there is little doubt in his own mind respecting the culpability of the mother. We grant that this is not the *beau ideal* of justice, and that it savours more of human frailty than of rigid impartiality in the judgment of right and wrong; but, as we have often said on former occasions, it is better that ten culprits should escape, than that one innocent person should suffer. If the unnatural mother do elude the vengeance of human laws, she cannot fly from a troubled conscience, more dreadful, because more permanent, than any punishment which an earthly tribunal can inflict.

Dr. Bernt, of Vienna, has lately proposed a new test respecting the vitality of new-born infants. This professor of medical jurisprudence enjoys the advantage of having the bodies of all the still-born children of the Lying-in-Hospital of Vienna at his disposal, the real state of whom, before death, can always be easily ascertained. We shall give the passage containing Dr. B's. test, in the words of our respected new cotemporary of the North.

"The circulation of a child undergoes an extensive revolution the moment it breathes, and the placental subsidiary action becomes suspended. The blood, arriving at the liver by the umbilical vein, is withdrawn from that organ, which ought to be rendered so much *lighter* by its abstraction; and the lungs, by the blood of the ductus arteriosus flowing through them, must have become proportionally heavier. The mere examination of these circumstances, in a new-born child, will go far to determine whether it has breathed; but the test which he chiefly rests upon, is the appearance and situation of the opening of the *foramen ovale*. *This hole, in a child which has never breathed, is exactly in the bottom of the fossa ovalis; but, as soon as the child has breathed, the aperture becomes turned towards the right*; in *several weeks*, it has ascended *very high*; and, in the adult age, is found to have arrived at the *summit of the oval*. In other words, from the moment respiration commences, the aperture of the foramen ovale begins to travel from the lower extremity of the oval hole towards the upper, proceeding from *left* to *right*; and the degree of advance it has made, becomes an index of the existence and duration of the respiratory process." 462.

The above test is considered as infallible by its author, and sincerely do we wish that it may prove so. On no occasion does a medical witness stand a greater hazard of loss of reputation, with almost certain laceration of feeling, than when giving evidence respecting the crime of infanticide. On this account we shall glance at one other docimasial test to which the German professor attaches much importance. This consists essentially in ascertaining the *relative gravity of the lung.* Thus, if the lung of a new-born infant, which generally weighs about an ounce and a half, *sink* in water, he observes how many grains, acting over a pulley, will just raise it to the surface. A lung of the weight above-mentioned may generally be raised by half a drachm. If the lung *swim in water,* he notices how much it takes to sink it. In lungs (if corresponding to the above, they weigh, when inflated, about six scruples more) he notes how many grains are necessary to sink them to the bottom. About 160 grains may be necessary for this purpose. He repeats these experiments with the heart attached to the lungs, and the whole of them a sufficient number of times to ascertain the relative proportion of these in an average way; and we have thus, he thinks, a steady hydrostatic test, sufficient to distinguish the steady relation of the inflated and fully circulating lung, to that which has never been fully permeated either by blood or air. For our own parts we do not see so clearly as the professor appears to do, the application of these last tests to the elucidation of the problem. But such as we have it we give it to our readers.

